# Surface Degradation of Mg_2_X-Based Composites at Room Temperature: Assessing Grain Boundary and Bulk Diffusion Using Atomic Force Microscopy and Scanning Electron Microscopy

**DOI:** 10.1021/acsami.4c10236

**Published:** 2024-08-28

**Authors:** Sanyukta Ghosh, Mohamed Abdelbaky, Wolfgang Mertin, Eckhard Müller, Johannes de Boor

**Affiliations:** 1 Institute of Materials Research, German Aerospace Center (DLR), Köln 51147, Germany; 2 Faculty of Engineering, Institute of Electronic Materials and Nanostructures (WET), University of Duisburg-Essen, Duisburg 47057, Germany; 3 Institute of Inorganic and Analytical Chemistry, JLU Giessen, Giessen 35390, Germany; 4 Faculty of Engineering, Institute of Technology for Nanostructures (NST), University of Duisburg-Essen, Duisburg 47057, Germany

**Keywords:** Mg_2_Si_1−*x*_Sn_*x*_, composite, atomic force microscopy, surface degradation, diffusion

## Abstract

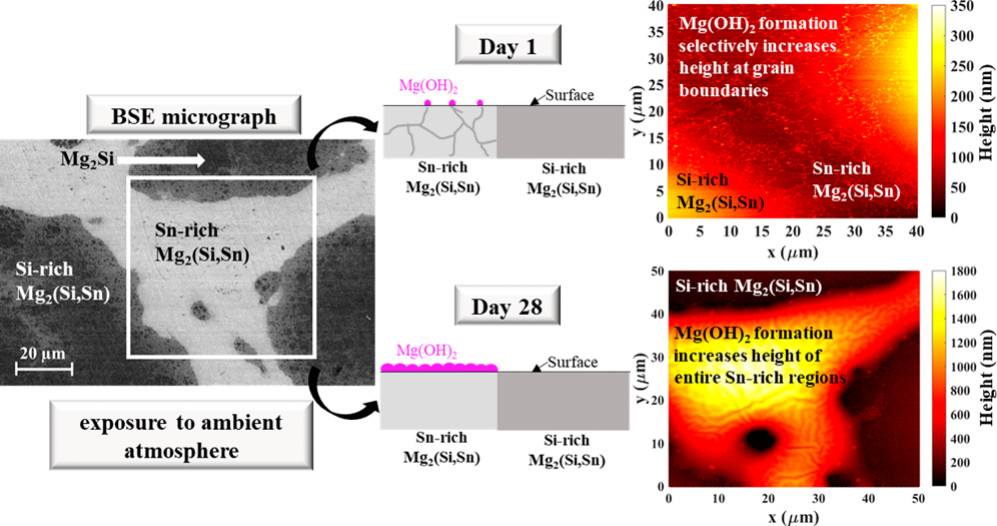

Practical application of thermoelectric generators necessitates materials that combine high heat-to-electricity conversion efficiency with long-term functional stability under operation conditions. While Mg_2_(Si,Sn)-based materials exhibit promising thermoelectric properties and module prototypes have been demonstrated, their stability remains a challenge, demanding thorough investigation. Utilizing atomic force microscopy (AFM) and scanning electron microscopy (SEM), we investigate the surface degradation of a composite material comprising Si-rich and Sn-rich Mg_2_(Si,Sn) solid solutions. The investigation reveals a pronounced dependence of stability on Sn content, with the Sn-rich phase Mg_2_Si_0.13_Sn_0.87_ displaying the formation of a nonprotective oxide layer. Subsequent AFM measurements provide evidence of dominating grain boundary diffusion of loosely bound Mg, compared to bulk diffusion, observed within a few days, ultimately resulting in a complete surface oxidation of the Sn-rich phase within several weeks. On the other hand, Mg_2_Si and Si-rich Mg_2_Si_0.80±0.05_Sn_0.20±0.05_ remain stable against Mg diffusion to the surface even after prolonged exposure. Comparison with previous investigations confirms that the degradation rate is found to be highly dependent on the Sn content, with markedly higher rates observed for *x* = 0.87 compared to *x* = 0.70 in Mg_2_Si_1–*x*_Sn_*x*_. These findings contribute to a better understanding of the stability challenges associated with Mg_2_(Si,Sn)-based materials, essential for the development of robust thermoelectric materials for practical applications.

## Introduction

The ability of thermoelectric (TE) materials to produce electrical power from otherwise wasted thermal energy has brought significant attention to this field, with immense potential for addressing critical issues such as energy efficiency, sustainable power generation, and environmental conservation.^1−3^ It offers advantages such as extremely high reliability, quiet operation, and the absence of moving parts, making it appealing for diverse applications from power generation to temperature sensing and cooling devices.^4−7^ The conversion efficiency of a TE generator is directly dependent on the performance of its constituent TE materials, which is quantified by the figure of merit: *zT* = *S*^2^σ/κ*T*, where *S* represents the Seebeck coefficient, σ denotes electrical conductivity, *T* signifies absolute temperature, and κ stands for thermal conductivity.^8^ Ongoing research in thermoelectricity endeavors to enhance thermoelectric properties, optimize device efficiency, increase power output, and discover new materials suitable for different applications across a wide temperature range. Furthermore, longevity, or durability, as demonstrated by several radioisotope thermoelectric generators (RTG) used in space missions, enduring over 40 years and more without failure, for example, in the Voyager, Cassini, and many other NASA and Russian space missions, is one of the advantages of TE technology and might compensate for moderate efficiencies.^9,10^ Consequently, stability at both the material and device levels emerges as a key requirement for their sustained practical application over time.

To date, numerous thermoelectric materials have become available for diverse applications across different operating temperatures.^11−15^ However, a notable drawback is that many of these materials contain toxic components. This poses potential environmental hazards during both handling and disposal processes. In this regard, magnesium silicide/magnesium stannide (Mg_2_*X*; *X* = Si, Sn)-based materials have emerged as compelling options. They offer high availability of raw materials, affordability, nontoxicity, and environmental friendliness, along with outstanding thermoelectric properties within the mid to high-temperature range (500–800 K).^16−18^

The solid solutions of Mg_2_Si and Mg_2_Sn have demonstrated notably enhanced thermoelectric properties compared to their binary counterpart, Mg_2_*X*. A remarkable *zT* value of 1.4 has been attained for n-type heavily doped Mg_2_Si_1–*x*_Sn_*x*_ (0.6 < *x* < 0.7) solid solutions, attributed to the degeneracy of the conduction bands and the reduction in lattice thermal conductivity due to alloy scattering.^19−21^ Correspondingly, for the p-type material, *zT* > 0.6 was achieved.^22,23^ To further enhance the thermoelectric properties, in addition to doping, energy filtering made possibly by spontaneous nanostructured composite formation can be employed.^24,25^ Furthermore, predictions suggest that Mg_2_(Si,Sn)-based thermoelectric generators (TEGs) could achieve conversion efficiencies surpassing 10%. Experimental efforts have realized conversion efficiencies ranging from 3% to 7% through the successful assembly of multiple Mg_2_(Si,Sn)-based modules, and Mg_2_(Si,Sn)/MgAgSb modules.^26−30^ However, practical implementation is impeded by material instability over prolonged operations.^31−37^

Mg_2_(Si,Sn) solid solutions with the most favorable thermoelectric properties often face thermodynamic instability, as their composition falls within the miscibility gap.^31,32^ Bourgeois et al.^33^ and Skomedal et al.^34^ investigated Mg_2_Si_1–*x*_Sn_*x*_ (*x* = 0.25, 0.6) and observed compositional stability up to 630 K, above which decomposition into multiple phases occurs. Søndergaard et al.^35^ observed rapid decomposition of Sn-rich phases upon heating to 673 K compared to Si-rich phases. Skomedal et al.^34^ also highlighted that Sn-rich solid solutions decompose more rapidly in oxygen containing atmosphere at lower temperatures compared to Si-rich ones. Apart from unmixing into multiple phases, Skomedal et al.^34^ and Yin et al.^37^ observed oxidation and formation of a nonprotective oxide layer upon heat treatment in air, with the oxidation onset temperature being highly dependent on Sn content. Zhang et al. studied the thermal stability of Mg_2_Si_1–*x*_Sn_*x*_ in an inert gas atmosphere, hoping to prevent oxidation formation.^36^ Observations by Zhang et al. revealed oxidation initiation in Mg_2_Si_0.4_Sn_0.6_(Sb) at around 700 K due to residual oxygen within inert gases, worsening above 800 K. Yin et al.^37^ investigated the stability of Mg_2_(Si_0.3_Sn_0.7_)_0.98_Sb_0.02_ in a vacuum and found severe Mg loss at 773 K through composition analysis using electron probe micro analyzer (EPMA). Such instability leads to the worsening of material properties and diminished device performance due to phase separation at elevated temperatures, particularly when Mg is lost, typically through sublimation owing to its high vapor pressure. Mg loss can occur during various synthesis stages and operational conditions, requiring synthesis and possibly operation adjustments. Interstitial Mg (I_Mg_^2+^) and Mg vacancies (V_Mg_^2–^) are two native point defects in solid solutions with relatively low defect formation energies.^38^ Mg sublimation enhances the density of V_Mg_^2–^. This Mg, which can be extracted from the material without decomposing the compound, is termed loosely bound Mg here. It easily diffuses out of the material due to the low vacancy formation energy and the low diffusion barrier.^38,39^ Moreover, it affects transport properties by trapping conduction electrons, as V_Mg_^2–^ are acceptor-type defects and can compensate for two electrons per defect. This phenomenon was further investigated by Kato et al.^40^ and Sankhla et al.^41^ They demonstrated that Mg loss significantly alters electronic transport properties in Mg_2_Si_1–*x*_Sn_*x*_, leading to decreased carrier concentration and consequently diminished transport properties, counteracting the intended doping. These studies demonstrate the complicated relationship between compositional and functional instability, both of which are closely intertwined with the Mg content.

The observed changes in material decomposition and transport properties in previous research underscore the stability challenges at higher temperatures. However, a very recent study by Duparchy et al.^39^ showed that not only at higher temperatures, but also when samples are stored at room temperature in ambient atmosphere for several years, selective surface degradation depending on the Sn content occurs. They linked the accelerated degradation of Sn-rich Mg_2_(Si,Sn) to the higher density of Mg vacancies in Mg_2_Sn compared to Mg_2_Si, as predicted by defect formation energies.^39^ They also reported changes in carrier concentration and transport properties due to the diffusion of loosely bound Mg from the bulk to the surface and subsequent oxidation. This alteration in Mg-based intrinsic defect concentrations contributes to the degradation of thermoelectric performance. However, as they observed the material after several years, it was not clear when exactly the sample degradation started, whether it is a gradual process unfolding over months or a rapid event occurring within days. Additionally, the initiation mechanism of degradation, whether through grain boundary diffusion or bulk diffusion of Mg, remains elusive despite previous attempts to understand it theoretically and experimentally.

In this work, we aim to understand these points. As composite materials offer an ideal platform for studying selective surface degradation and oxidation we conducted an investigation using a composite sample comprising proportions of Si-rich and Sn-rich phases. We employed atomic force microscopy (AFM) to examine (changes in) surface due to exposure to air. Since AFM measures the relative height changes of different phases, it is essential to have a reference phase within the same sample that exhibits minimal or no height change over time. Given that Mg_2_Si has been shown to be stable in our previous study,^39^ performing AFM on a composite sample containing Si-rich, Sn-rich, and Mg_2_Si phases will allow us to measure the height change of the Sn-rich phase relative to Mg_2_Si, using Mg_2_Si as a reference. Subsequently, we conducted scanning electron microscopy (SEM) combined with energy-dispersive X-ray (EDX) analysis on the degraded surface to correlate AFM analysis with the composition. Our findings revealed that oxidation selectively occurring in the Sn-rich phase led to the formation of a nonprotective oxide layer. It initiated through the diffusion of loosely bound Mg to grain boundaries and subsequent oxidation within a few days, before spreading across the entire Sn-rich region via bulk diffusion within a month.

## Experimental Section

Starting elements Mg (turnings, Merck), Si (<6 mm, Chempure) and Sn (<71 μm, Merck) with purity >99.5% were weighed according to stoichiometric ratios and then ball-milled using a SPEX 8000D Shaker high-energy ball mill to prepare Mg_2_Si and Mg_2_Sn. To compensate for Mg loss during the synthesis and sintering processes, an excess of 2 at. % Mg was added, resulting in nominal compositions of Mg_2.06_Si and Mg_2.06_Sn. A composite comprising binary Mg_2.06_Si and Mg_2.06_Sn in a 50:50 atomic ratio was synthesized by milling the respective powders for 6 min, followed by sintering under a vacuum pressure of 10^–5^ bar, applying a pressure of 66 MPa for a duration of 10 min at a temperature of 873 K. The sintered pellets were polished using a series of sandpapers ranging from lower to higher grades, followed by diamond polishing powder, to achieve a smooth surface finish. Subsequently, the samples were stored for 1 week in a vacuum-sealed plastic bag, which maintained a rough vacuum environment. After this period, the samples were removed from the plastic bag, and topography measurements using an atomic force microscope (AFM) were conducted on the sample surface. We used a Bruker Innova AFM system in noncontact mode. The probes used are platinum silicide tips (PTSi-FM) from Nanosensors with a resonance frequency of around 75 kHz. The initial measurement, referred to as day 1 measurement, served as the reference point for subsequent analyses. Following the day 1 measurement, the samples were stored in ambient atmosphere conditions, and AFM measurements were performed over various time spans spanning several weeks. These subsequent measurements were conducted on day 4, day 28 (4 weeks), and day 35 (5 weeks) postinitial measurement. The timing of all measurements was calculated relative to the day 1 measurement. After 50 days of exposure to ambient atmosphere conditions, scanning electron microscopy (SEM) was used to assess surface degradation. Secondary electron (SE) images, backscattered electron (BSE) images, and elemental maps of the constituent elements were acquired using a Zeiss Ultra 55 SEM equipped with energy-dispersive X-ray (EDX) spectroscopy (PentaFETx3). To facilitate comparison between degraded and nondegraded surfaces, the sample surface was slightly polished to remove the oxide layer, and SEM imaging was conducted. This step allowed for a direct comparison of surface features before and after exposure to ambient atmosphere conditions.

## Results

The composite exhibited distinct regions, characterized by a Sn-rich region (bright area) and a Si-rich region (black colored area), as shown in Figure S1, in agreement with our previous work.^24^ Within the Si-rich region, the presence of (binary) Mg_2_Si particles was also observed. The phenomenon of solid solution formation, instead of the coexistence of Mg_2_Si and Mg_2_Sn binaries, as well as the composition of the distinct phases formed, has been extensively explained in reference.^24^ The composition of the Sn-rich region was Mg_2_Si_0.13_Sn_0.87_, while the Si-rich region exhibited an average composition of ∼Mg_2_Si_0.80±0.05_Sn_0.20±0.05_. SEM and AFM analyses were conducted on a composite sample containing both Sn-rich and Si-rich solid solutions to examine surface topography changes over time, with a focus on understanding both the surface oxidation mechanism and selective oxide formation depending on the Sn content.

**Figure 1:**
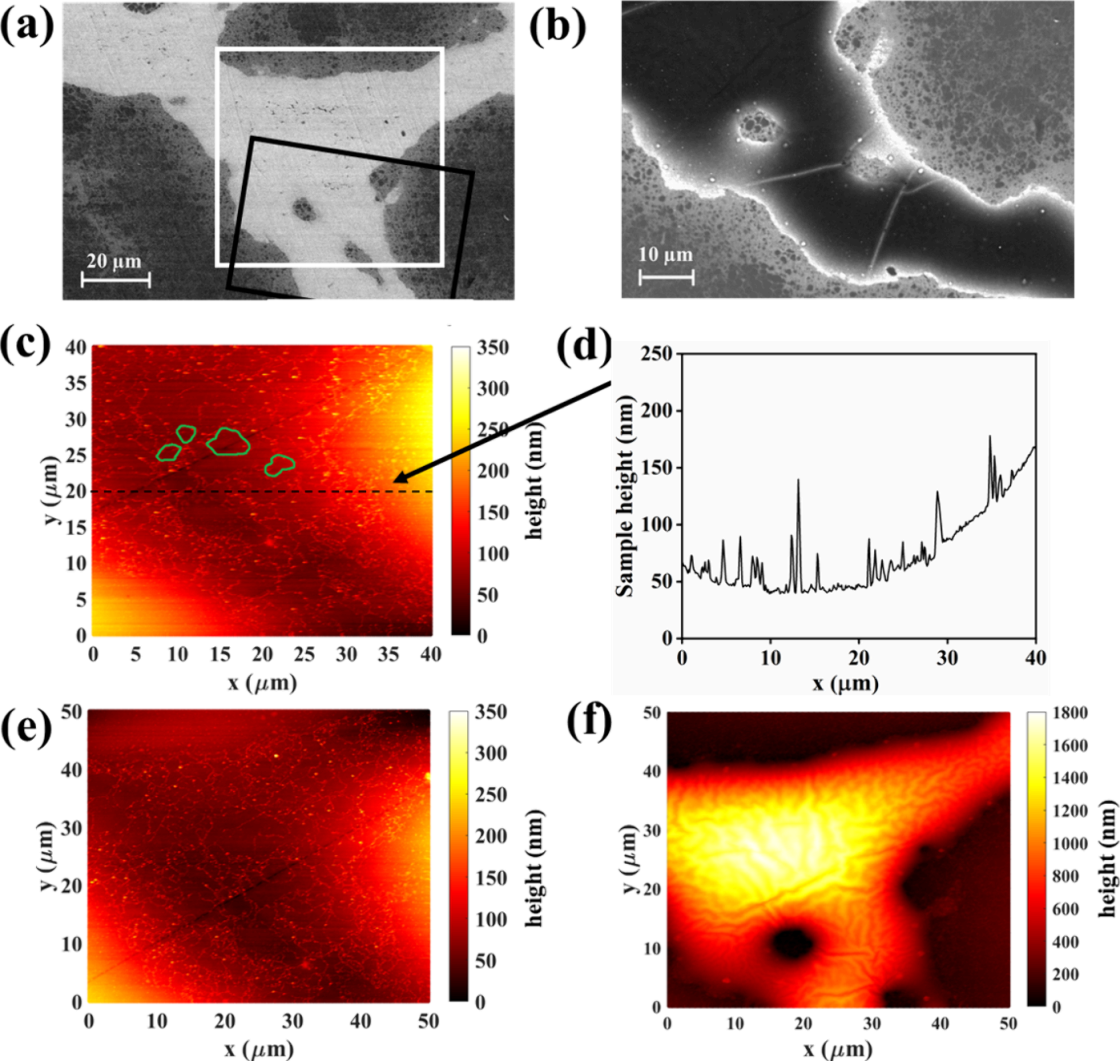
BSE micrographs of the of the Si-rich/Sn-rich composite showing (a) the fresh surface after polishing and (b) the degraded surface after 50 days of storage in an ambient atmosphere (region marked by the black rectangle in (a)). The region marked by a white rectangle in (a) was subjected to AFM analysis at various time points: (c) day 1 (64% of the marked region), (e) day 4, and (f) day 35. (d) The line scan of the sample height, taken from (c) along the indicated black line. A few grains in (c) are marked with green color.

Figure 1(a) depicts the BSE micrograph capturing the polished surface of the composite after aging, and (b) illustrates the BSE micrograph of the degraded surface after 50 days of storage in ambient atmosphere. Initially, in the BSE micrograph, the Sn-rich matrix appear brighter, while the Si-rich islands appear darker, consistent with expectations due to the higher atomic mass of Sn. However, following several weeks of exposure to air, the Sn-rich region became darker than the islands, indicating the formation of a surface layer comprising lower mass elements than the Si-rich region. To understand these changes with time and how the formation of the surface layer is initiated, AFM surface topography of a similar region was captured over a time span of several weeks and is shown in Figure 1 (c-e).

Figure 1(c) presents the AFM topography image acquired on day 1, revealing that the Si-rich region exhibited greater elevation compared to the Sn-rich counterpart. Furthermore, elevated height was observed along the grain boundaries within the Sn-rich region on day 1, making the grains distinctly visible. The grain sizes were approximately within the range of 3–7 μm (Figure 1(c)). The observed grain boundary size closely aligns with those reported in the literature for Sn-rich solid solutions synthesized via the ball-milling route. A grain size of 4–6 μm was reported for Mg_2_Si_0.4_Sn_0.6_,^42^ and an average grain size of around 5 μm was reported for Mg_2_Si_0.2_Sn_0.8_,^43^ consistent with our findings. The line scan depicting the height of the sample along the indicated line in Figure 1(c) is presented in Figure 1(d). The height increase at the grain boundaries in the Sn-rich matrix was roughly 40 nm. Conversely, clear grain boundaries were not evident within the Si-rich region. Subsequent AFM measurements conducted on day 4 (Figure 1(e)) showcased similar topography, with increased elevation observed along the grain boundaries within the Sn-rich region.

Given the absence of significant differences in topography observed over the course of several days (between days 1 and 4), AFM analysis was repeated on day 28 (week 4) and day 35 (week 5). The measurements on day 28 and day 35 (Figure 1(f)) revealed a substantial change in the height of the Sn-rich region, suggesting the formation of a surface layer. Additionally, apart from changes in height, a vein-like structure was observed on the sample surface of the Sn-rich region (Figure 1(f)). Subsequent measurements on day 28 and day 35 showed no height difference in the topography of the Sn-rich region, suggesting minimal changes over this time period. On the other hand, there was no observable topographic change in the Si-rich region, implying minimal alterations in the Si-rich phase. Thus, assuming negligible height change in the Si-rich island, we can infer a height increase of approximately 1.5 μm in the Sn-rich region, indicative of the formation of a nonprotective surface layer with a thickness of 1.5 μm. Consequently, after 28 days of storage in air, Si-rich islands exhibited lower height compared to the Sn-rich region, despite initially having higher elevations.

**Figure 2:**
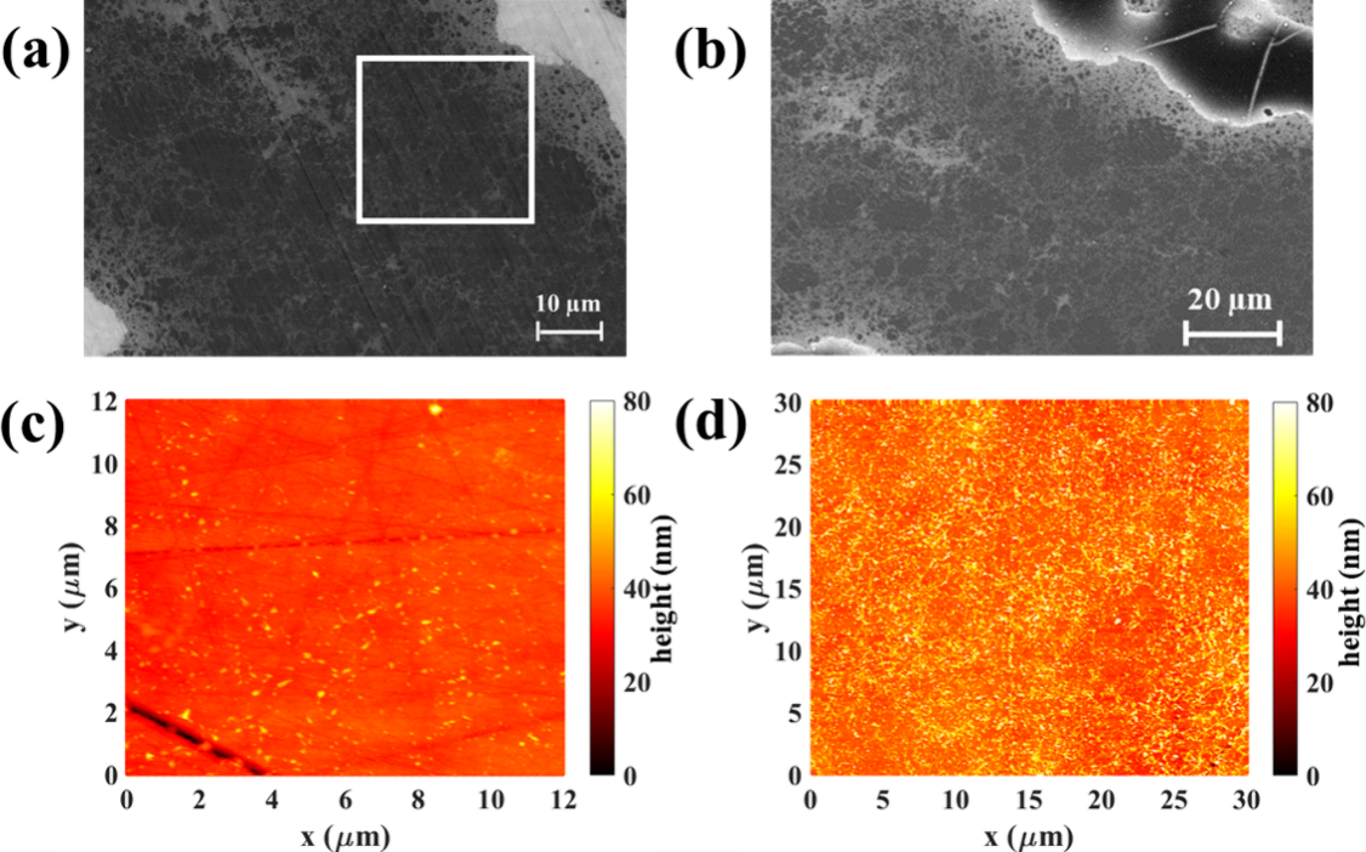
BSE micrographs taken from a Si-rich island of the Si-rich/Sn-rich composite showing (a) the freshly polished surface and (b) the degraded surface after 50 days of storage in ambient atmosphere. The approximate area within the marked region in (a) was subjected to AFM analysis at (c) 1 day and (d) 35 days.

Since Figure 1 shows minimal changes in the Si-rich island phase, to conduct a detailed investigation, AFM and SEM micrographs were also captured with higher resolution at the Si-rich island phase over a span of a few weeks, as shown in Figure 2. The position of the region under investigation with respect to that in Figure 1 is defined in Figure S1. Figure 2 (a) and (b) present BSE micrographs of the polished surface of the aged sample and the surface after 50 days of storage in air, respectively. Upon examining the two BSE micrographs, no changes in color contrast are observed, suggesting negligible changes in the Si-rich regions due to aging. For further clarification, AFM measurements were conducted in the same region. Although our intention was to focus on the exact region of the Si-rich island for AFM measurements each time, slight variation in position is inevitable due to the necessity of sample mounting for each measurement. The measurement on day 1 (Figure 2(c)) showed mostly uniform topography, despite spatial variations in the Si content evident in the BSE micrograph. No grain boundary decoration was observed in the Si-rich phase, unlike in the Sn-rich phase. The same topography was observed in the measurements performed on day 4. The measurement on day 35, as shown in Figure 2(d) exhibited some substructure and an increased roughness, that might be related to the variations in Si content, but further investigations are required to substantiate this point. Furthermore, scratches appeared less prominent in the day 35 measurements (Figure 2(d)) compared to day 1 and day 4. Overall, it can be concluded that changes in the surface topology within the Si-rich phases over this time frame are minimal or at least much weaker than those observed in the Sn-rich regions.

**Figure 3:**
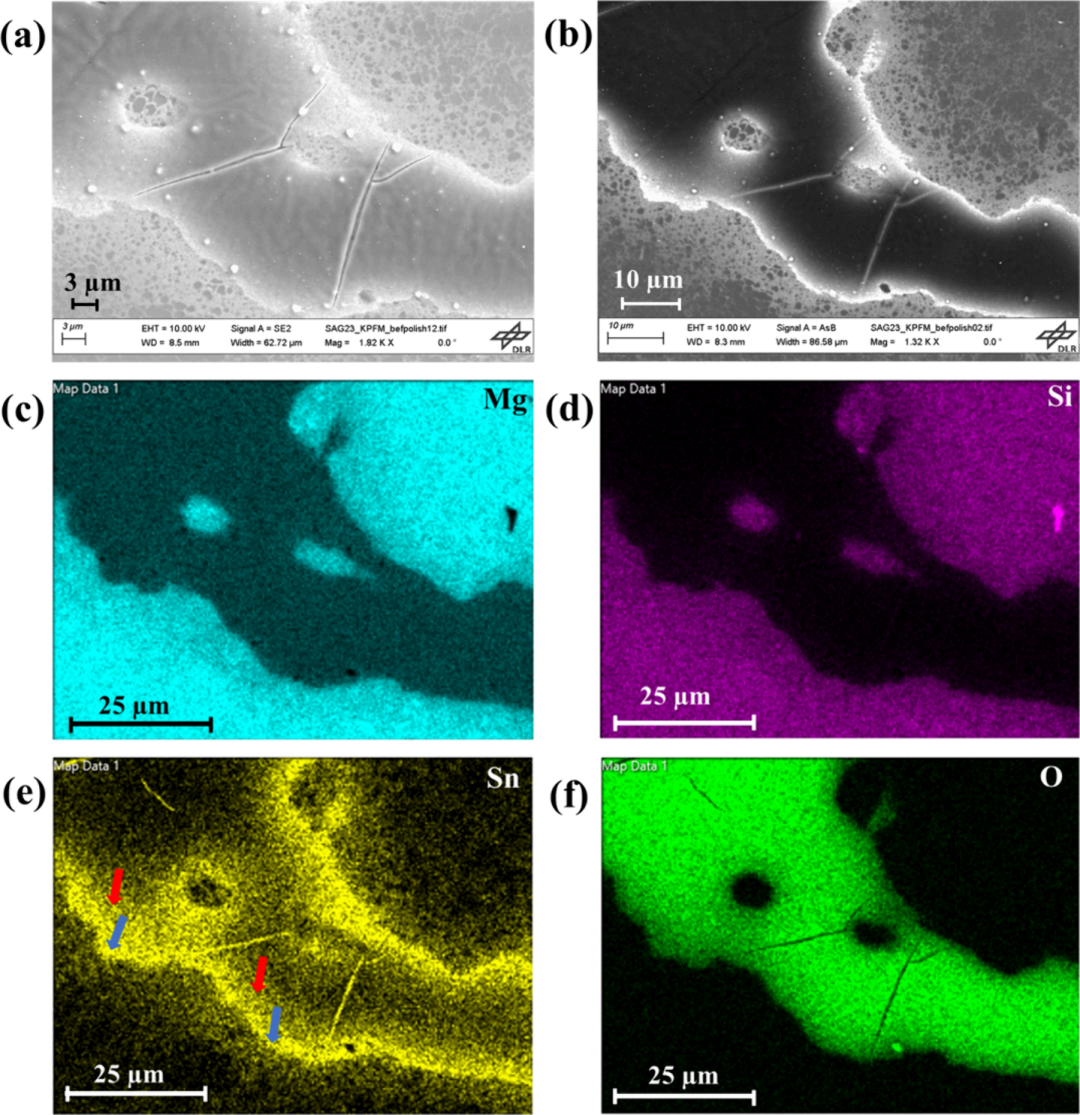
(a) SE micrograph, (b) BSE micrograph, and the corresponding elemental mapping images of (c) Mg, (d) Si, (e) Sn (one diffusively smeared margin at the interfaces: the outer boundary is marked by blue arrow and the inner boundary is marked by red arrow), and (f) O of the Si-rich/Sn-rich composite after 50 days of exposure to air.

Figure 3 shows SE and BSE images alongside the corresponding elemental maps of the same region. Detailed SEM analysis conducted on the aged sample following AFM observation, revealed the exclusive presence of an oxide layer covering the Sn-rich region (Figure S2). This finding aligns with the increased height profile observed in the Sn-rich region after exposure to air for 28 days, as observed in AFM measurements. This phenomenon is attributed to the reaction of loosely bound Mg with moisture at the surface of the Sn-rich region, leading to the formation of an Mg(OH)_2_ layer.^39,44^ Although detecting hydrogen via EDX analysis is unfeasible, our inference of a Mg(OH)_2_ layer rather than a MgO layer stems from the observation that the surface darkened during aging (as seen in Figure S3), whereas MgO typically appears white^45^ and necessitates higher temperatures for formation.^46^ Furthermore, the lower Gibbs free energy of formation of Mg(OH)_2_ (Δ*G*_*f*_^Mg(OH)2^ = −834 kJ/mol) compared to MgO (Δ*G*_*f*_^MgO^ = −569 kJ/mol) at 298 K^47^ suggests that in the presence of ambient humidity, loosely bound Mg would favor the formation of Mg(OH)_2_ over MgO, as described by the equation:
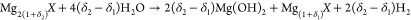
1where (δ_2_ – δ_1_) is the loosely bound Mg.

There is either no or very little oxygen present in the Si-rich phases, as observed from the elemental map. To investigate the observed substructure (Figure 2(d)) in the Si-rich region, EDX point scan analysis was performed on both Si-rich and Sn-rich regions, as shown in Table 1. This showed a very low oxygen concentration in the Si-rich region of the aged sample, a concentration typically observed even in freshly polished samples. Consequently, the underlying cause of the observed substructure in the Si-rich region remains ambiguous. It is possible that this subtle alteration could stem from dust agglomeration, although further investigation is necessary to clarify this aspect definitively. Elevated Sn content was notably observed at the interfaces of the Sn-rich and Si-rich regions and at the observable cracks in the amorphous oxide layer (Figure S4). This observation suggests that cracks in the oxide layer expose underlying Sn content. At the interfaces, there is a margin with a sharp border at the domain boundary adjacent to the Si-rich material, while a diffusively smeared transfer region extends into the Sn-rich domain. The outer sharp boundary is due to a slightly higher Sn content in the TE material, whereas the inner one corresponds to the thinning oxide surface layer toward its edges (Figure 3(e)). Similarly, a faint inner margin was observed for Si within the Sn-rich region at the interfaces, as shown in Figure S5(e). Furthermore, a few spherical structures were also observed on the surface, and elemental mapping (Figure S5) confirmed their composition as predominantly Sn-rich, with traces of magnesium and oxygen also detected (Figure S5). This differs from the observation by Duparchy et al.,^39^ where primarily Mg(OH)_2_ spherical structures were found on the surface of n-type Mg_2.06_Si_0.3_Sn_0.665_Bi_0.035_ after a year of air exposure. Notably, their sample had a different composition and was not a composite.

**Table 1:** Composition (Atom %) of Si-Rich Phase and Sn-Rich Phase on the Freshly Polished Surface and Degraded Surface of the Composite from EDX Analysis

	Freshly polished surface				Degraded surface after 50 days of storage			
Phase	Mg	Si	Sn	O	Mg	Si	Sn	O
Sn-rich phase	63.5	4.9	28.6	3.0	22.1	1.2	6.5	70.2
Si-rich phase	65.9	28.3	5.0	0.9	64.3	24.6	7.4	3.7

## Discussion

A composite synthesized by mixing 50% Mg_2_Si and 50% Mg_2_Sn displayed two distinct regions postsintering, a Sn-rich region and a Si-rich island with varying Si content. The Si-rich island phase also contained small Mg_2_Si particles.

Upon AFM analysis conducted on the first day of measurement, the surface topography revealed a notable difference in height between the Si-rich and Sn-rich phases, despite thorough polishing efforts aimed at achieving a mirror-like finish (Figure 1). The Si-rich phase exhibited a significantly higher elevation compared to the Sn-rich phase, with an observed height difference of approximately 200 nm. Such discrepancies in surface height between composite phases are not uncommon in mechanically polished samples, owing to the different hardness characteristics of the constituent materials. Soft materials typically undergo rapid removal during polishing, resulting in a comparatively lower height compared to the harder components. Previous research has demonstrated higher hardness in Mg_2_Si or Si-rich phases compared to the Mg_2_Sn or the Sn-rich solid solution, attributable to the strong covalent bonding between silicon and magnesium atoms,^48−50^ thereby explaining the observed height difference and elevated height of the Si-rich phase.

AFM measurements on both day 1 and day 4 showed a slight increase in grain boundary height within the Sn-rich phase, approximately around 40 nm (Figure 1(d)), indicating that oxidation initiated primarily at the grain boundaries. Duparchy et al. found a decrease in carrier concentration for an n-type Mg_2.06_Si_0.3_Sn_0.665_Bi_0.035_ sample stored in ambient atmosphere for several years, indicating diffusion of Mg out of the grains through lattice diffusion, with Mg vacancies compensating for electron concentration in n-type materials.^39^ However, it remained uncertain whether Mg migrates to the next grain boundary and then undergoes grain boundary diffusion, or primarily diffuses by lattice diffusion. Typically, diffusion rates differ between grain boundaries and the bulk crystalline lattice, with bulk diffusion being relatively slow and grain boundary diffusion much more rapid at temperatures significantly lower than the melting point, effectively serving as a short circuit path for the transport of atoms alongside bulk diffusion.^51,52^ In this case, the elevated height where the grain boundaries meet the sample surface suggests that loosely bound Mg first arrives at the surface via grain boundaries. It then accumulates at these boundaries, where it reacts with moisture, initiating Mg(OH)_2_ formation at these sites. Subsequently, the overall increase in the height of the Sn-rich region confirms hydroxide formation starting from the grain boundary and spreading across the entire Sn-rich region after exposure of 28 days. The complete surface coverage may result from a combination of transport from the inner along grain boundaries and lateral diffusion parallel to the surface by bulk diffusion in the Mg_2_X. Notably, there was no significant difference observed between the measurements at 28 and 35 days, indicating that once the entire surface had undergone oxidation, further changes are minimal with time.

Conflicting findings exist regarding the diffusion mechanism in Mg_2_(Si,Sn). Wang et al.^53^ and Kogut et al.^54^ conducted investigations on Mg_2_Si growth. Wang et al.^53^ concluded that during Mg_2_Si growth, bulk diffusion and phase reaction predominate over grain boundary diffusion. In contrast, Kogut et al.^54^ demonstrated a mixed diffusion phenomenon involving bulk diffusion and grain boundary diffusion, with the relative influence depending on temperature. Specifically, during the initial stages of Mg_2_Si growth on thin films, lattice interstitial Mg diffusion into the Si substrate primarily drives the transport mechanism. As temperature rises, grain boundaries become increasingly influential, accelerating diffusion. It is worth noting that the diffusion mechanisms may differ between Mg_2_Si and its solid solutions. Additionally, diffusion in films can differ from bulk diffusion as films might be strained, altering diffusion barriers. Yasseri et al.^31^ conducted diffusion experiments on Mg_2_Sn/Mg_2_Si at 600 °C and concluded that material transport involves both melt infiltration and diffusion homogenization, with rapid grain boundary diffusion alongside slower bulk diffusion, forming the observed “cauliflower” structure of Si-rich phase. However, solid proof of grain boundary diffusion was lacking. Recently, Duparchy et al.^39^ investigated the stability of Mg_2.06_Si_0.3_Sn_0.665_Bi_0.035_ materials, synthesized via melting process, exposed to air for several years and found uniform coverage by a nonprotective layer of Mg(OH)_2_ across the surface, without variation at grain boundaries. This contradicts the expectation that dominating grain boundary diffusion would result in preferential decoration of Mg(OH)_2_ at grain boundaries. This discrepancy may arise because the transition from grain boundary coverage to complete surface coverage by Mg(OH)_2_ layer is a rapid process, already completed at the time of characterization. Additionally, Bi doping may play a role, as it can increase vacancy concentration, facilitating bulk transport parallel to the sample surface.

Detecting grain boundary diffusion requires high-resolution techniques due to the extremely small numbers of atoms involved. Previous attempts to observe grain boundary diffusion using SEM analysis on materials exposed to air for a few days were unsuccessful, likely due to the very thin oxide layer formation at grain boundaries. This observation is consistent with our findings, where the height of the nonprotective layer at the grain boundary is approximately ∼40 nm after exposure to air for 1 day. Our study provides evidence supporting the assertion that oxidation primarily initiates at grain boundaries and progresses rapidly, reaching full surface coverage within a few weeks.

The increase in surface height by 1.5 μm over a period from 4 days to 28 days indicates the formation of a surface layer of the same thickness. Such a thick layer of Mg(OH)_2_ necessitates a considerable amount of Mg. Consequently, over time, there must be diffusion from within the depth of the sample, rather than only a surface effect. This observation aligns with the changes in bulk TE properties noted by Duparchy et al. during prolonged storage at room temperature, despite the removal of the surface layer through polishing.^39^ Sankhla et al. estimated that the amount of loosely bound Mg, the removal of which can facilitate the transition from a Mg-rich to a Mg-poor state without material decomposition, is approximately δ_2_ – δ_1_ = 0.004 for Mg_2_Si_0.385_Sn_0.6_Sb_0.015_.^41^ By applying this value to eq 1, a rough estimation suggests that Mg emerges from a depth of approximately 190 μm within the sample to form the 1.5 μm thick Mg(OH)_2_ layer. The minimal difference between measurements at day 28 and day 35 implies that after the full surface coverage, subsequent changes are minimal. This is expected as the formation of Mg(OH)_2_ continues until all loosely bound Mg is oxidized. However, as the layer thickness increases, the process slows due to longer diffusion paths through the oxide. This is in accordance with the regular square root law of diffusion-controlled grain growth.

Comparing the results of this work with the little previous work on the topic we can deduce that the reaction speed increases with increasing tin content in the solid solution: Duparchy et al.^39^ demonstrated an oxide layer thickness of 700 nm for Mg_2.06_Si_0.3_Sn_0.665_Bi_0.035_, after a 6 month exposure. In contrast, we find an oxide layer thickness of 1.5 μm for a composition of Mg_2_Si_0.13_Sn_0.87_, after only 28 days of exposure to air. However, it remains uncertain whether this difference in oxide layer thickness solely stems from the varying Sn content or if the presence of Bi dopant also contributes to the observed effects. Additionally, Duparchy et al.^39^ observed the formation of Mg(OH)_2_ spherical structures at the surface of the Sn-rich regions. In our study, we observed a thick, continuous, and mostly uniform oxide layer, which might be a subsequent stage of spherical structure formation, as depicted in Figure 3, resulting in a vein-like structure on the sample surface.

Not observing significant alteration in the topography of Si-rich regions over exposure time confirms the selective oxidation of Mg_2_(Si,Sn) materials, which depends on the Sn content in agreement with the findings of Duparchy et al.,^39^ Skomedal et al.^34^ and Søndergaard et al.^35^ Duparchy et al. also found no oxide formation on the Mg_2_Si phase even after 2 years of storage.^39^ Similarly, in our study, we speculate that the red-colored region in Figure 2(c-d), corresponds to the Mg_2_Si phase, while the yellow-colored region, which exhibited a 20 nm height increase after 35 days, corresponds to the Mg_2_Si_0.80±0.05_Sn_0.20±0.05_ phase covered with hydroxide. However, further investigations are necessary to support this hypothesis. The oxidation is minimal to the extent that it was not detected in SEM-EDX analysis (Figure 3).

Mg_2_Si and the Si-rich Mg_2_Si_0.80±0.05_Sn_0.20±0.05_ phase remain stable even after 50 days of exposure. At the moment, it looks like the upper limit of the stability range aligns with the boundaries of the miscibility gap, although this cannot be confirmed without further experimentation. Mg_2.06_Si_0.3_Sn_0.665_Bi_0.035_ exhibited a surface layer thickness of 700 nm after 6 months, whereas measurements on Mg_2_Si_0.13_Sn_0.87_ indicate formation of a 1.5 μm thick surface layer after 28 days. This suggests that stability follows a strongly nonlinear trend, with degradation rate escalating rapidly with increasing Sn content. The underlying reason for this behavior remains unclear, although one possibility is the effect of Bi dopant, and another is variations in the Mg diffusion coefficient and defect concentration within the material corresponding to different Sn content. According to the calculations, the Mg diffusion coefficient in Mg_2_Si is higher than in Mg_2_Sn, but the defect concentration is higher in Mg_2_Sn that in Mg_2_Si.^39^ The diffusion coefficient might be higher for solid solutions due to a larger degree of disorder, possibly stemming from a reduced diffusion barrier caused by lattice strain. The quantity of loosely bound Mg may vary across materials with different Sn content. DFT defect formation energy calculations predict an increasing amount of loosely bound Mg with increasing Sn content^38^ but elemental data on this is scarce and with significant uncertainties: Kato et al.^40^ estimated δ_2_ – δ_1_ to be 0.003 for Mg_2_(Si_0.5_Sn_0.5_)_1–*x*_Sb_*x*_, while Sankhla et al.^41^ estimated δ_2_ – δ_1_ to be 0.004 for Mg_2_Si_0.385_Sn_0.6_Sb_0.015_. Although the difference is marginal, it could potentially be higher for Mg_2_Si_0.13_Sn_0.87_, resulting in the formation of a thicker surface oxide layer.

Figure 3 and Figure S2 reveal elevated measured Sn content at the interfaces of Si-rich and Sn-rich phases, as well as within observed cracks in the Mg(OH)_2_ phase after 50 days of exposure to air. However, this does not necessarily indicate a higher concentration of Sn in these specific regions. Rather, the presence of cracks in the oxide layer exposes the underlying Sn content, giving the appearance of a higher concentration over the whole area of the domain. While Si is also present in these regions, its lower concentration in the Sn-rich phase renders it less visibly distinct. Furthermore, as the interfaces serve as the lateral boundaries of the oxide layer, Sn content also appears in high concentration at the interfaces due to the absence of an oxide layer. The observed cracks in the oxide layer were not visible in AFM maps, hence they developed between the day 35 to day 50 timeline. One possible explanation for the formation of cracks in the oxide layer could be the limited diffusion of Mg through Mg(OH)_2_. If Mg cannot diffuse through Mg(OH)_2_, then new Mg(OH)_2_ must form at the old surface by water diffusion. The formation of new Mg(OH)_2_ beneath the existing layer might cause a volume change, leading to internal stresses and potentially resulting in cracks.

Additionally, along with these cracks, a few spherical structures were predominantly observed at the interfaces of Sn-rich and Si-rich phases (Figure 3 and Figure S5), rich in Sn content, with traces of Mg and O. Duparchy et al.^39^ observed Mg(OH)_2_ spherical structures throughout the surface of Sn-rich Mg_2.06_Si_0.3_Sn_0.665_Bi_0.035_ phase, but in our case, we know that the thickness is larger and we speculate this is because of next stage in film formation.

Coating the material surface with an appropriate barrier substance can effectively prevent the formation of Mg(OH)_2_, as demonstrated in various studies.^34,36,37^ Recent research by Ying et al. has shown that atomic layer deposition on Mg-based modules significantly reduces Mg(OH)_2_ formation for Mg_3_(Sb, Bi)_2_ based compounds, thereby enhancing durability and stability under practical operating conditions.^55^ The degradation of this material is not exclusively influenced by the Sn content but also by the Mg content.^39^ Nonetheless, irrespective of the Mg content, it is evident that the Si-rich phase exhibits greater stability compared to the Sn-rich region. However, the highest performing material is Sn-rich, specifically, Mg_2_Si_1–*x*_Sn_*x*_ with *x* = 0.6–0.7. Hence, there seems to be a trade-off between performance and stability. Nevertheless, research on Si-rich solid solutions has revealed reasonably high *zT* values^56,57^ suggesting that with further optimization, also the Si-rich phase may hold promise for practical applications.

## Conclusions

The study comprehensively investigated the surface topography of a composite material formed by mixing binary Mg_2_Si and Mg_2_Sn and sintering at 600 °C, using AFM and SEM techniques. The material consisted of Si-rich Mg_2_Si_0.80±0.05_Sn_0.20±0.05_ and Sn-rich Mg_2_Si_0.13_Sn_0.87_ solid solutions. Our findings underscore the critical role of Sn content on sample stability, with Sn-rich phases exhibiting pronounced instability, noticeable already after a few weeks of storage under ambient conditions, evidenced by layer growth readable from an elevated surface in the Sn-rich regions compared to neighboring Si-rich domains. Moreover, AFM measurements revealed alterations in surface topography along the grain boundary lines of the Sn-rich phase after only a few days of atmospheric exposure, suggesting accelerated grain boundary diffusion of loosely bound Mg compared to bulk diffusion, ultimately leading to complete surface oxidation of Sn-rich regions. Conversely, Si-rich phases remain stable after several weeks of exposure. SEM-EDX analysis verified these findings by revealing the presence of oxygen solely on the Sn-rich phase areas, with minimal to negligible oxygen detected on the Si-rich phase. Diffusion mechanisms, notably influenced by temperature and material composition, were found to significantly contribute to the degradation process, with higher concentrations of Sn accelerating degradation rates. Based on our finding that grain boundary diffusion is more relevant, future research can focus on suppressing grain boundary diffusion in Mg_2_X as a strategy to reduce material degradation to an acceptable level, potentially providing a more feasible alternative to controlling lattice diffusion.

## Supporting Information Available

BSE micrograph of the freshly polished surface of the Si-rich/Sn-rich Mg_2_(Si, Sn) composite (Figure S1); SEM micrograph and the corresponding elemental mapping images of the composite following 7 weeks of air exposure (Figure S2); optical microscope images of the composite at day 1 and day 28 of storage in air (Figure S3); SEM micrograph captured from a zoomed-in region of the composite, primarily illustrating fractures on the oxide layer and spherical structures at the interfaces after 7 weeks of exposure to air (Figure S4); BSE micrograph, along with corresponding elemental mapping images, illustrating the presence of spherical structures by the yellow dashed circles on the surface of the composite after 7 weeks of exposure to air (Figure S5) (PDF)
