# Supplementary material for: Surface Degradation of Mg2X-Based Composites at Room Temperature: Assessing Grain Boundary and Bulk Diffusion Using Atomic Force Microscopy and Scanning Electron Microscopy
Source: ACS Appl Mater Interfaces. 2024 Aug 28;16(36):48619–28. doi: 10.1021/acsami.4c10236 (PMC11403557; doi:10.1021/acsami.4c10236)
Supplement: Supplementary file 1 — am4c10236_si_001.pdf [file am4c10236_si_001.pdf]

Supporting Information

Surface Degradation of Mg_2X-Based Composites at Room Temperature: Assessing Grain Boundary and Bulk Diffusion using Atomic Force Microscopy and Scanning Electron Microscopy

Sanyukta Ghosh[1], Mohamed Abdelbaky[2], Wolfgang Mertin[2], Eckhard Müller[1,3], Johannes de Boor[1,4]**

[1]Institute of Materials Research, German Aerospace Center (DLR), Köln, Germany
[2]University of Duisburg-Essen, Faculty of Engineering, Institute of Electronic Materials and Nanostructures (WET), Duisburg, Germany
[3]JLU Giessen, Institute of Inorganic and Analytical Chemistry, Giessen, Germany
[4]University of Duisburg-Essen, Faculty of Engineering, Institute of Technology for Nanostructures (NST), Duisburg, Germany
*E-mail of the corresponding author: sanyukta.ghosh@dlr.de, Johannes.deboor@dlr.de

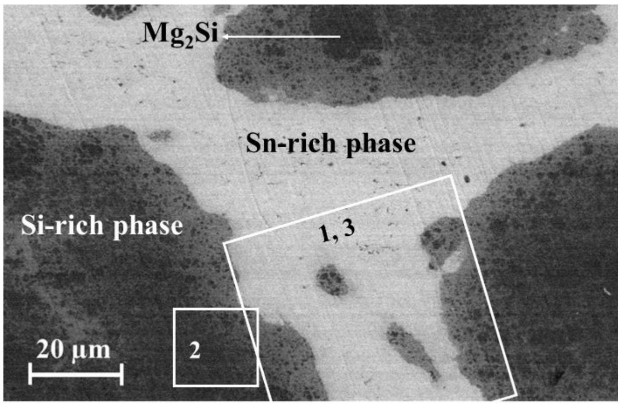

Figure S1. BSE micrograph of the freshly polished surface of the Si-rich/Sn-rich $Mg_2(Si, Sn)$ composite. The boxes labeled 1, 2 (approximate area) and 3 indicate the corresponding regions referenced in Figures 1, 2 and 3 in the main text.

The composite consisted of Si-rich $Mg_2(Si, Sn)$, Sn-rich $Mg_2(Si, Sn)$, and Mg_2Si particles. AFM measurements were performed within the square regions labeled 1 and 2, with the corresponding data displayed in Figures 1 and 2, respectively, in the main text. Furthermore, SEM analysis was conducted in region 1 to correlate topographical changes with composition, as depicted in Figure 3 of the main text.

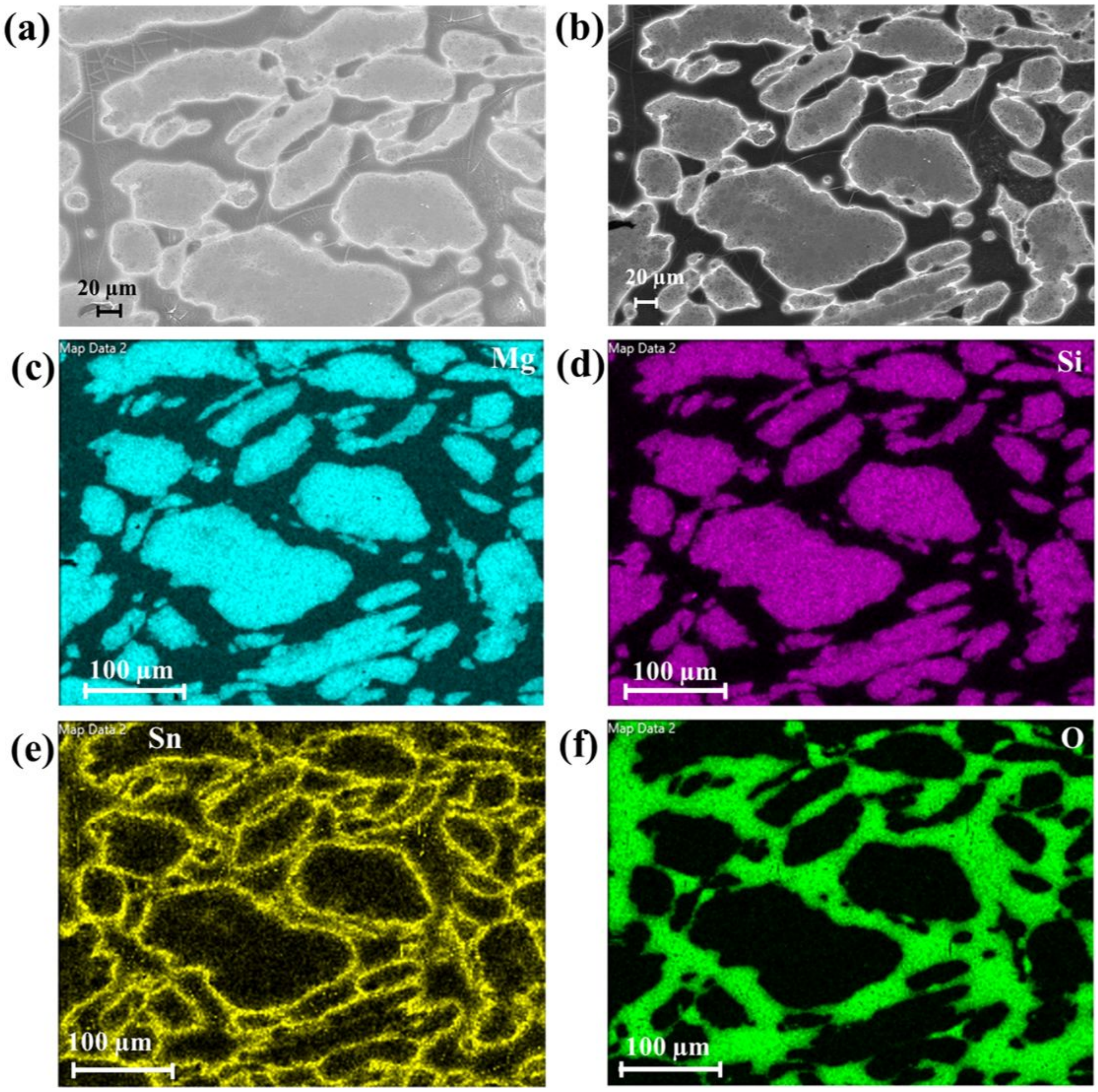

Figure S2. (a) SE micrograph, (b) BSE micrograph, and the corresponding elemental mapping images of (c) Mg, (d) Si, (e) Sn and (f) O across a wide area of the Si-rich/Sn-rich Mg_2(Si, Sn) composite following 7 weeks of air exposure.

SE and BSE micrographs captured on the surface of a composite aged for 50 days, consisting of Si-rich and Sn-rich Mg_2(Si, Sn) solid solutions, revealed selective surface degradation of Sn-rich regions. This degradation was evident from changes in topography and color contrast. The presence of more oxygen in the Sn-rich regions further verified this observation.

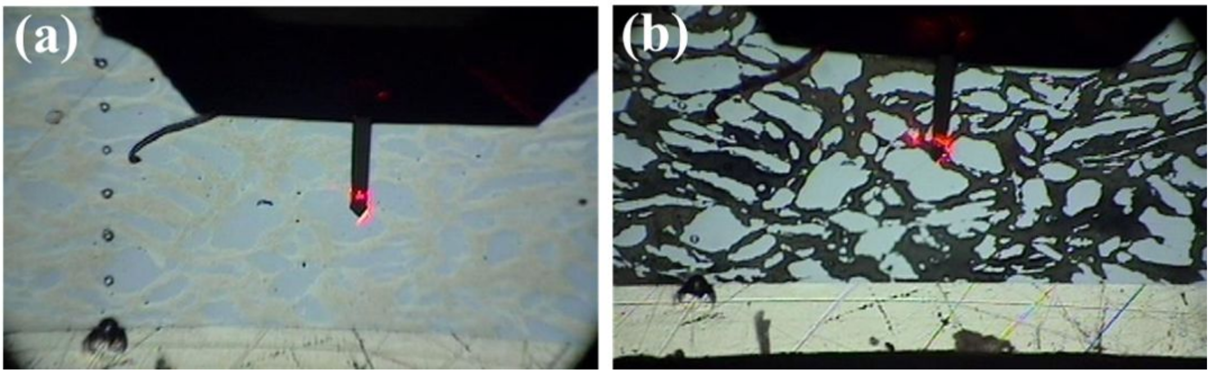

Figure S3. Optical microscope images of the Si-rich/Sn-rich $Mg_2(Si, Sn)$ composite at (a) day 1 and (b) day 28 of storage in air.

Optical microscope images of the composite showed that the Si-rich islands remained visually unchanged even after 28 days of exposure to air. However, the Sn-rich regions turned black from grey, indicating the formation of a non-protective layer selectively on the Sn-rich areas after 28 days of exposure.

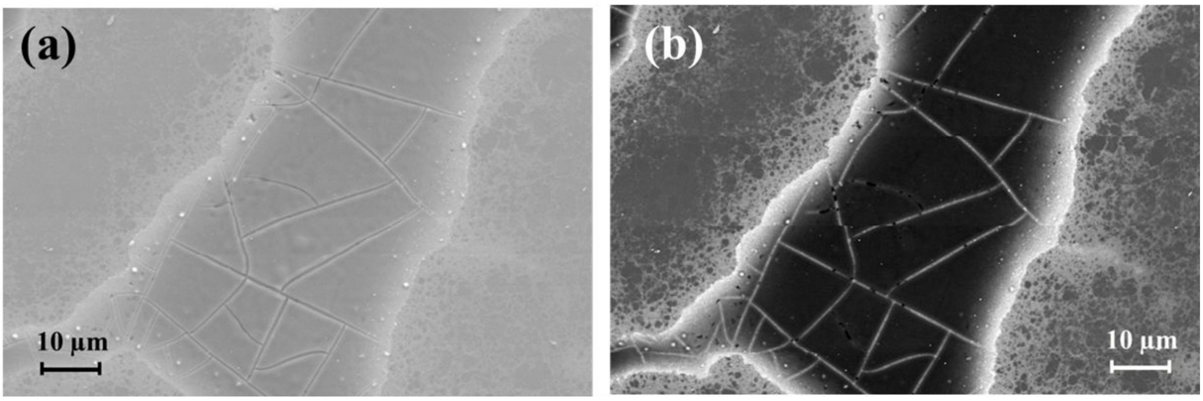

Figure S4. (a) SE micrograph, and (b) BSE micrograph captured from a zoomed-in region, primarily illustrating fractures on the oxide layer and spherical structures at the interfaces of the Si-rich/Sn-rich $Mg_2(Si, Sn)$ composite after 7 weeks of exposure to air.

Figure S4(a) shows the presence of cracks in the oxide layer on the Sn-rich region after 50 days of storage in air. The BSE micrograph confirms that the fracture has a higher atomic density than the oxide layer, indicating the Sn-rich phase beneath. This observation is discussed in detail in the main text.

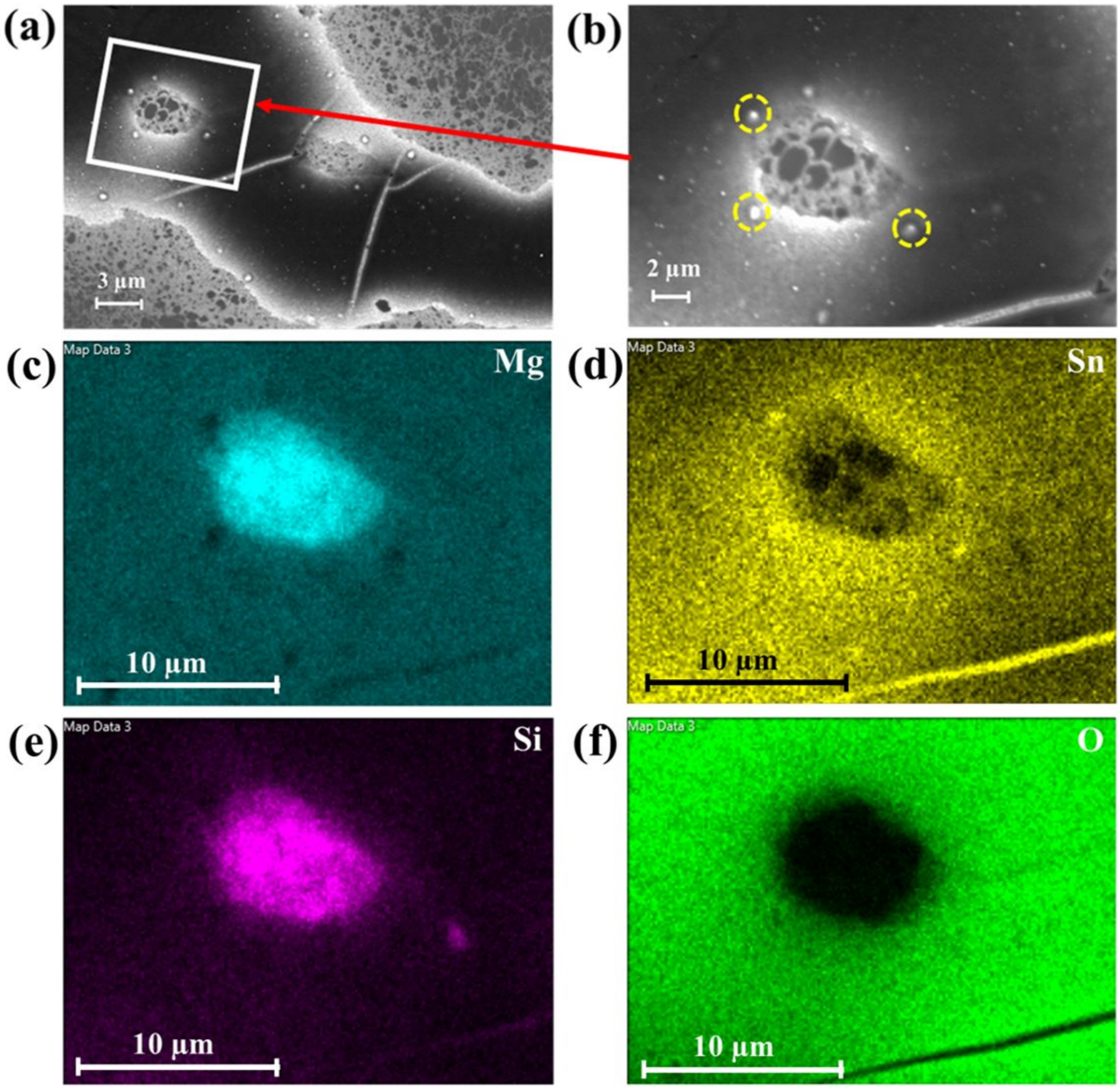

Figure S5. (a) and (b) BSE micrograph, along with corresponding elemental mapping images of (c) Mg, (d) Sn, (e) Si, and (f) O captured from a zoomed-in region, primarily illustrating the presence of spherical structures by the yellow dashed circles on the surface of the Si-rich/Sn-rich Mg_2(Si, Sn) composite after 7 weeks of exposure to air.

Figure S5 shows spherical structures predominantly located at the interfaces between Si-rich and Sn-rich phases. These structures were found to be primarily Sn-rich, with small amounts of magnesium and oxygen.
